# Supplementary material for: Deep Learning Methodologies Applied to Digital Pathology in Prostate Cancer: A Systematic Review
Source: Diagnostics (Basel). 2023 Aug 14;13(16):2676. doi: 10.3390/diagnostics13162676 (PMC10453406; doi:10.3390/diagnostics13162676)
Supplement: Supplementary file 1 [file diagnostics-13-02676-s001.zip › Supplementary Figure S1.pdf]

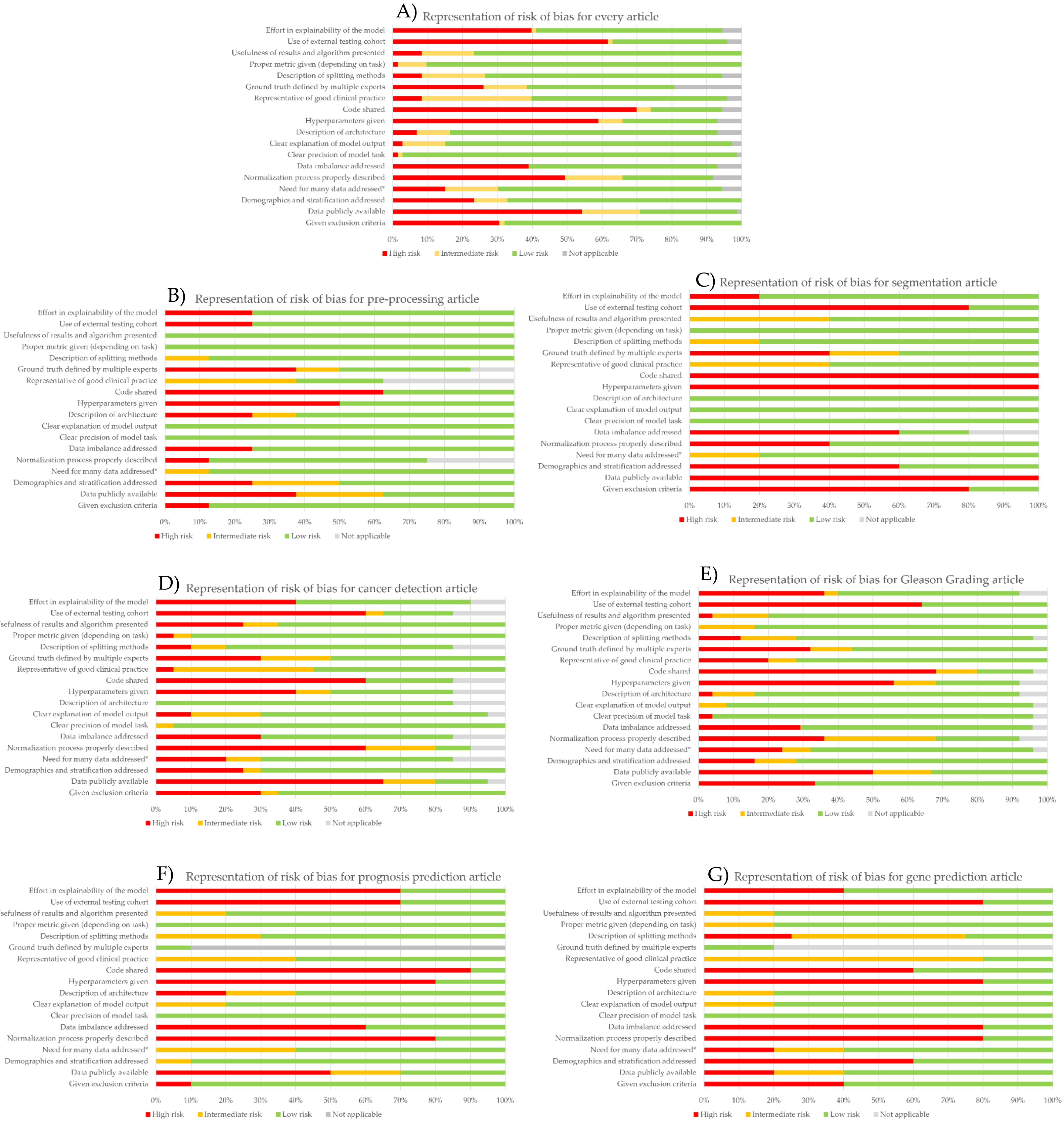

Supplementary Figure S1. Representation of Risk of Bias for subsets of selected articles. A) All selected articles. B) Articles focusing on pre-processing. C) Articles focusing on segmentation. D) Articles focusing on cancer detection. E) Articles focusing on Gleason Grading. F) Articles focusing on prognosis prediction. G) Articles focusing on gene prediction
