# Supplementary material for: Deep Learning Methodologies Applied to Digital Pathology in Prostate Cancer: A Systematic Review
Source: Diagnostics (Basel). 2023 Aug 14;13(16):2676. doi: 10.3390/diagnostics13162676 (PMC10453406; doi:10.3390/diagnostics13162676)
Supplement: Supplementary file 1 [file diagnostics-13-02676-s001.zip › Supplementary Table S1_RoB_revised_final.pdf]

| Question / Article ref | Existence of exclusion criteria | Data publicly available | Reference to demographics and stratification | Sufficient amount of training data | Normalization process properly described | Strategy for data imbalance | Description of model task | Description of model output | Description of architecture | Definition of hyperparameter values | Code publicly available | Ground truth in accordance with good clinical practice | Ground truth defined by multiple experts | Description of splitting methods | Definition of a metric appropriate to the analysis | Relevance of algorithm with state of the art | Use of external testing cohort | Explainability of the model |
|---|---|---|---|---|---|---|---|---|---|---|---|---|---|---|---|---|---|---|
| Pre-processing | | | | | | | | | | | | | | | | | | |
| [16] | | | | | | | | | | | | NA | | | | | | |
| [17] | | | | | NA | | | | | | | | | | | | | |
| [18] | | | | | | | | | | | | NA | | | | | | |
| [19] | | | | | | | | | | | | | | | | | | |
| [20] | | | | | | | | | | | | | | | | | | |
| [22] | | | | | NA | | | | | | | NA | NA | | | | | |
| [23] | | | | | | | | | | | | | | | | | | |
| [24] | | | | | | | | | | | | | | | | | | |
| [25] | | | | | | | | | | | | | | | | | | |
| Segmentation | | | | | | | | | | | | | | | | | | |
| [26] | | | | | | NA | | | | | | | | | | | | |
| [29] | | | | | | | | | | | | | | | | | | |
| [28] | | | | | | | | | | | | | | | | | | |
| [30] | | | | | | | | | | | | | | | | | | |
| [27] | | | | | | | | | | | | | | | | | | |
| Cancer detection | | | | | | | | | | | | | | | | | | |
| [43] | | | | | | | | | | | | | | | | | | |
| [44] | | | | | | | | | | | | | | | | | | |
| [45] | | | | | | | | | | | | | | | | | | |
| [46] | | | | | | | | | | | | | | | | | | |
| [32] | | | | | | | | | | | | | | | | | | |
| [55] | | | | | | | | | | | | | | | | | | |
| [50] | | | | | | | | | | | | | | | | | | |
| [56] | | | | | | | | | | | | | | | | | | |
| [31] | | | | | | | | | | | | | | | | | | |
| [34] | | | | | | | | | | | | | | | | | | |
| [38] | | | | | | | | | | | | | | | | | | |
| [51] | | | | | | | | | | | | | | | | | | |
| [57] | | | | | | | | | | | | | | | | | | |
| [33] | | | | | | | | | | | | | | | | | | |
| [49] | | NA | | NA | | NA | | NA | NA | NA | NA | | | NA | | | NA | |
| [48] | | | | NA | NA | NA | | | NA | NA | NA | | | NA | | | NA | NA |
| [47] | | | | NA | NA | NA | | | NA | NA | NA | | | NA | | | NA | NA |
| [58] | | | | | | | | | | | | | | | | | | |
| [53] | | | | | | | | | | | | | | | | | | |
| [54] | | | | | | | | | | | | | | | | | | |
| [59] | | | | | | | | | | | | | | | | | | |
| Gleason grading | | | | | | | | | | | | | | | | | | |
| [63] | | | | | | | | | | | | | | | | | | |
| [74] | | | | | | | | | | | | | | | | | | |
| [21] | | | | | | | | | | | | | | | | | | |
| [64] | | | | | | | | | | | | | | | | | | |
| [73] | | | | | | | | | | | | | | | | | | |
| [52] | | | | | | | | | | | | | | | | | | |
| [65] | | | | | | | | | | | | | | | | | | |
| [78] | | | | | | | | | | | | | | | | | | |
| [66] | | | | | | | | | | | | | | | | | | |
| [77] | | | | | | | | | | | | | | | | | | |
| [72] | | | | | | | | | | | | | | | | | | |
| [61] | | | | | | | | | | | | | | | | | | |
| [35] | | | | | | | | | | | | | | | | | | |
| [36] | | | | | | | | | | | | | | | | | | |
| [37] | | | | | | | | | | | | | | | | | | |
| [39] | | | | | | | | | | | | | | | | | | |
| [62] | | | | | | | | | | | | | | | | | | |
| [79] | | | | | | | | | | | | | | | | | | |
| [75] | | | | NA | NA | NA | | | NA | NA | NA | | | NA | | | | NA |
| [40] | | | | | | | | | | | | | | | | | | |
| [76] | | | | | NA | | NA | NA | NA | NA | | | | | | | | NA |
| [68] | | | | | | | | | | | | | | | | | | |
| [69] | | | | | | | | | | | | | | | | | | |
| [71] | | | | | | | | | | | | | | | | | | |
| [40] | | | | | | | | | | | | | | | | | | |
| [70] | | | | | | | | | | | | | | | | | | |
| [67] | | | | | | | | | | | | | | | | | | |
| Prediction (prognosis and genomic signatures) | | | | | | | | | | | | | | | | | | |
| [81] | | | | | | | | | | | | | NA | | | | | |
| [83] | | | | | | | | | | | | | NA | | | | | |
| [88] | | | | | | | | | | | | | NA | | | | | |
| [87] | | | | | | | | | | | | | NA | | | | | |
| [84] | | | | | | | | | | | | | NA | | | | | |
| [85] | | | | | | | | | | | | | NA | | | | | |
| [82] | | | | | | | | | | | | | NA | | | | | |
| [86] | | | | | | | | | | | | | NA | | | | | |
| [80] | | | | | | | | | | | | | NA | | | | | |
| [42] | | | | | | | | | | | | | | | | | | |
| [92] | | | | | | | | | | | | | NA | | | | | |
| [89] | | | | | | | | | | | | | NA | | | | | |
| [91] | | | | | | | | | | | | | | | | | | |
| [93] | | | | | | | | | | | | | NA | | | | | |
| [90] | | | | | | | | | | | | | NA | | | | | |

**Supplementary Table S1.** Details of risk of bias for all articles. Green: low risk, yellow: intermediate risk, red: high risk. NA: Not Applicable.
