# Supplementary material for: Deep Learning Methodologies Applied to Digital Pathology in Prostate Cancer: A Systematic Review
Source: Diagnostics (Basel). 2023 Aug 14;13(16):2676. doi: 10.3390/diagnostics13162676 (PMC10453406; doi:10.3390/diagnostics13162676)
Supplement: Supplementary file 1 [file diagnostics-13-02676-s001.zip › Supplementary Table S2_metrics_table.pdf]

Metric	Application	Definition
True positive	Detection	An event of interest that exists and is correctly predicted.
True negative	Detection	Absence of an event of interest that is correctly predicted.
False positive	Detection	An event of interest that is predicted but does not exist.
False negative	Detection	An event of interest that exists but is not predicted.
Accuracy (ACC)	Segmentation<br>Classification	Ratio between the sum of true positives and true negatives and the total number of events of interest. It is not a reliable metric when working with unbalanced data, which is often the case in pathomics.<br>Ranges from 0 to 1 (best performance)
Sensitivity/Recall (Sens)	Classification	Ratio between the true positives and the sum of true positives and false negatives. A high sensitivity/recall implies that most events are correctly predicted. It can be high even if many events are wrongfully predicted.<br>Ranges from 0 to 1 (best performance).
Specificity (Spec)	Classification	Ratio between the true negatives and the sum of true negatives and false positives. A high specificity implies that very few events are wrongfully predicted. It can be high even if most events are missed.<br>Ranges from 0 to 1 (best performance).
Precision	Classification	Ratio between the true positives and the sum of true positives and false positives. A high precision implies that most detected events are correctly predicted. It can be high even if most events are missed.<br>Ranges from 0 to 1 (best performance).
False positive rate	Detection	Ratio between false positives and the sum of false positives and true negatives.<br>Ranges from 0 to 1 (best performance).
F1 score	Segmentation<br>Classification	Ratio between twice the multiplication of precision and recall and the addition of precision and recall.<br>Ranges from 0 to 1 (best performance)
ROC-AUC (AUC)	Segmentation<br>Classification<br>Prediction	ROC AUC is the Area Under the Curve (AUC) of the Receiver Operating Characteristic (ROC). ROC is obtained by using increasing thresholds on predicted probabilities to define sensitivity/recall and false positive rate.<br>Ranges from 0 to 1 (best performance)
Jaccard/Intersection over Union (IoU)	Segmentation	Ratio between the area of overlapping predicted and ground truth masks divided by the area of combined predicted and ground truth masks.<br>Ranges from 0 to 1 (best performance).

Dice coefficient	Segmentation	Ratio between twice the area of overlapping predicted and ground truth masks and the sum of the areas of predicted masks and ground truth masks.<br>Ranges from 0 to 1 (best performance).
Cohen's Kappa coefficient (Kappa)	Classification<br>Prediction	Statistic between two raters comparing their agreement to the agreement that would occur by chance. Cohen's Kappa coefficient is used to measure agreement between pathologists as well as agreement between a model and a pathologist.<br>Ranges from 0 to 1 (best performance).
Weighted or Quadratic Cohen's Kappa coefficient ((q)wKappa)	Classification<br>Prediction	Cohen's Kappa coefficient that penalizes disagreement by multiplying with a weight that is increasing when disagreement is getting larger. The quadratic Cohen's kappa coefficient corresponds to a weighted Cohen's kappa coefficient with a squared penalization.
Pearson's Correlation Coefficient (PCC)	Segmentation<br>Prediction	Covariance of two variables divided by the product of their standard deviations.<br>If two variables never coincide, their PCC is equal to -1. If two variables are identical, their PCC is equal to 1. If there is no linear dependency between two variables, their PCC is equal to 0.
Odds Ratio (OR)	Prediction	Ratio of the odds for one event to happen in the presence of a second type of event and the odds for the same event to happen in the absence of the second type of event. This statistic is symmetric and is used to evaluate the correlation between two different events.<br>Odds ratio superior to 1 implies that the two events are correlated. Odds ratio inferior to 1 implies that the two events are anti-correlated. Odds ratio equal to 1 implies that the two events are independent.
Hazard ratio (HR)	Prediction	Ratio of the hazard rates between groups of patients. Most often, hazard corresponds to death or response to a treatment.<br>Hazard ratio is usually depicted using Kaplan-Meier curves.

Supplementary Table S2. Metrics found in articles of the review and their definition
