# Supplementary material for: Deep Learning Methodologies Applied to Digital Pathology in Prostate Cancer: A Systematic Review
Source: Diagnostics (Basel). 2023 Aug 14;13(16):2676. doi: 10.3390/diagnostics13162676 (PMC10453406; doi:10.3390/diagnostics13162676)
Supplement: Supplementary file 1 [file diagnostics-13-02676-s001.zip › Supplementary Tables_S3-to-S8_for_revised_final.pdf]

Supplementary Table S3. Papers focusing on pre-processing tasks.

First author, year Reference	DL architecture(s)	Methodology for/to	Ground-truth	Training cohort	Internal validation (IV)	External validation (EV)	Aim	Results
Schömig-Markiefka *et al*, 2021 [16]	InceptionResNetV2	Quality assessment	Tumor, glandular and non-glandular prostate tissue annotations	Already trained	Subset of TCGA slides	686 WSIs	Impact of artifacts for tissue classification	Tissue classification performance decreases with appearance of artifacts
Haghighat *et al*, 2022 [17]	ResNet18 PathProfiler	Quality assessment	3 pathologists' assessment of usability (unanimity)	198 WSIs	3819 WSIs	None	Prediction of tile-level usability<br>Prediction of slide level usability	AUC: 0.94 (IV)<br>AUC: 0.987 (IV)<br>PC: 0.889 (IV)
Brendel *et al*, 2022 [18]	ResNet34-IBN	Quality assessment	Purity score (TCGA), pathologist' purity score (WCM)	4984 WSIs TCGA (6 cancer types)	866 WSIs TCGA (6 cancer types)	78 WSIs	Cancer and tissue type prediction<br>Tumor purity cutoff 70%	F1: 1 (IV)<br>F1: 0.83 (EV)<br>AUC: 0.8 (IV)
Anghel *et al*, 2019 [19]	VGG-like network	Staining normalization	Cancer presence annotation	96 WSIs	14 WSIs	None	Improve cancer detection when using poor quality WSI	F1 (base): 0.79<br>F1 (best): 0.87
Otàlora *et al*, 2019 [20]	MobileNet [20] + 2 layers	Staining normalization	Gleason patterns 3 and 4 presence (but not annotation)	3540 TMAs	1321 TMAs	3961 TMAs	Impact of normalization for GP classification	AUC (base): 0.56 (IV) & 0.48 (EV)<br>AUC (best): 0.84 (IV) & 0.69 (EV)<br>F1 (base): 0.56 (IV) & 0.48 (EV)<br>F1 (best): 0.67 (IV) & 0.62 (EV)
Rana *et al*, 2020 [22]	GANs	Virtual staining	Manually stained H&E slides<br>Non-stained slides<br>Manual annotation of tumor areas and grades made by 4 pathologists	148 WSIs	13 WSIs	None	Comparison of dye and computationally-stained images<br>Comparison of unstained and computationally destained images<br>Comparison of annotations between dye and computationally-stained WSIs	$\overline{PC}$: 0.96<br>Structural similarity index: 0.9<br>$\overline{PC}$: 0.96<br>Structural similarity index: 0.9<br>$\overline{IoU}$ tumor: 0.79<br>$\overline{IoU}$ healthy: 0.9
Sethi *et al*, 2016 [23]	Custom CNN	Staining normalization	Annotations of glands at pixel level	20 WSIs	10 WSIs	None	Epithelial-stromal segmentation	AUC: 0.96 (Vahadane)<br>AUC: 0.95 (Khan)
Swiderska-Chadaj *et al*, 2020 [24]	U-Net GANs	Staining normalization	Cancer, benign and other tissue type annotation Slide labels of cancer presence	324 WSIs	258 WSIs	85 WSIs<br>50 WSIs	Impact of normalization for cancer detection	AUC: 0.92<br>AUC: 0.98 (GAN)<br>AUC: 0.83<br>AUC: 0.97 (GAN)

Salvi *et al*, 2021 [25]	Inceptionv3 pretrained on ImageNet	Improve performances when using stain normalization and tile selection	Pathologist classification of benign and tumoral cases	400 WSIs	100 WSIs	None	Tile level classification adding normalization (norm) then tile selection (all)	Sens (base): 0.82<br>Spec (base): 0.57<br>Sens (norm): 0.74<br>Spec (norm): 0.83<br>Sens (all): 0.95<br>Spec (all): 0.90
							WSI level classification adding normalization (norm) then tile selection (all)	Sens (base): 0.94<br>Spec (base): 0.68<br>Sens (norm): 0.78<br>Spec (norm): 0.95<br>Sens (all): 1<br>Spec (all): 0.98

IV: Internal Validation. EV: External Validation. TCGA: The Cancer Genome Atlas project. TMA: Tissue Micro Array. CNN: Convolutional Neural Network. GP: Gleason Pattern
AUC: Area Under the Curve. PC: Pearson Correlation. F1: F1-score. $\overline{metric}$ implies the mean of this metric (e.g. $\overline{PC}$)

Supplementary Table S4. Papers focusing on segmenting glands or tissue to help future classification tasks

First author, year Reference	DL architecture(s)	Methodology for/to	Ground-truth	Training cohort	Internal validation (IV)	External validation (EV)	Aim	Results
Ren *et al*, 2017 [26]	U-Net	Segment glands	Manual annotations of glands	22 WSIs	5-fold cross val	None	Gland segmentation in H&E slides	F1: 0.84
Li *et al*, 2019 [29]	R-CNN	Segment epithelium	Hand annotations by a consensus of pathologists	40 patients	5-fold cross val	None	Epithelial cell detection	AUC: 0.998
Bulten *et al*, 2019 [28]	U-Net	Segment glands	Binary epithelium mask, corrected by 3 non experts	20 WSIs	5 WSIs	None	Epithelium segmentation based on IHC	IoU: 0.854<br>F1: 0.915
Bukowy *et al*, 2020 [30]	SegNet (VGG16)	Segment Stroma, Epithelium, Lumen (SEL)	Annotation of SEL by pathologists (HG) or by machine (MG)	10 WSIs	6 WSIs	None	Segment and compare training annotations (HG or MG)	$\overline{AUC}$: 0.89 (MG)<br>$\overline{AUC}$: 0.85 (HG)<br>$\overline{AUC}$: 0.93 (MG fine-tuned HG)
				140 WSIs			Epithelium segmentation with a combination of the 3 models	$\overline{ACC}$: 0.86 (DL)<br>$\overline{ACC}$: 0.77 (only MG)

Salvi *et al*, 2021 [27]	U-Net + post-processing	Segment glands	Manual annotations by 2 pathologists	100 patients	50 patients	None	Gland segmentation	Dice: 0.901 (0.892 only U-Net)

IV: Internal Validation. EV: External Validation. CNN: Convolutional Neural Network. HG: Human Generated. MG: Machine Generated.
AUC: Area Under the Curve. F1: F1-score. IoU: Intersection Over Union. $\overline{metric}$ implies the mean of this metric. cross val: cross-validation.

Supplementary Table S5. Papers focusing cancer detection only

First author, year Reference	DL architecture(s)	Methodology for/to	Ground-truth	Training cohort	Internal validation (IV)	External validation (EV)	Aim	Results
Litjens *et al*, 2016 [43]	Custom CNN	Cancer detection	Annotation by 3 pathologists	150 patients	75 patients	None	Cancer detection on pixel	AUC: 0.99
Kwak *et al*, 2017 [44]	Custom CNN	Cancer detection	Disease status by 1 pathologist	162 TMAs	491 TMAs	None	Cancer detection on sample	AUC: 0.974
Kwak *et al*, 2017 [45]	Custom CNN	Cancer detection	Pathology report	162 TMAs	185 TMAs	None	Cancer detection on sample	AUC: 0.95
Campanella *et al*, 2018 [46]	ResNet34 or VGG11--BN (MIL principle)	Cancer detection	Pathology report	12610 WSIs	1824 WSIs	None	Cancer detection at WSI level	AUC: 0.98
Campanella *et al*, 2019 [32]	RNN (MIL)	Cancer detection	Pathology report	12 132 WSIs	1784 WSIs	12 727 WSIs	Cancer detection at WSI level (RNN)	AUC: 0.991 (IV) 0.932 (EV)
	ResNet34 (MIL)						Cancer detection at WSI level (ResNet)	AUC: 0.986 (IV)
Garcià *et al*, 2019 [55]	VGG19	Gland malignancy detection	Gland malignancy pixel annotation by 1 pathologist	6195 glands (from 35 WSIs)	5-fold cross val	None	Malignancy gland classification	AUC: 0.889
Singh *et al*, 2019 [50]	ResNet22	Cribriform pattern detection	Cribriform presence annotated by 1 pathologist	749 WSIs	3-fold cross val	None	Cribriform pattern detection at tile level	ACC: 0.88
Jones *et al*, 2019 [56]	ResNet50 & SqueezeNet	Cancer detection	Tile malignancy, evaluated by pathologists	1000 tiles from 10 WSIs	200 tiles	70 patches (Google search then reviewed)	Malignancy detection at tile level	$\overline{ACC}$: 0.96 (IV) 0.78 (EV)

Duong *et al*, 2019 [31]	ResNet50 and multiscale embedding	Cancer detection with multiscale approach	Pathologist defined disease status	602 TMAs	303 TMAs	None	TMA classification using only x10 magnification	AUC: 0.961
							TMA classiciation with multi scale embedding	AUC: 0.971
Raciti *et al*, 2020 [38]	PaigeProstate	Cancer detection	Diagnosis made by genitourinary pathologists with ancillary studies	Already trained (Campanella)	Already trained (Campanella)	232 biopsies	Malignancy classification of WSIs	Sens: 0.96<br>Spec: 0.98
							Improvement of pathologist's patient classification	$\overline{Sens}$: 0.738 (path)<br>0.900 (w/ AI)<br>$\overline{Spec}$: 0.966 (path)<br>0.952 (w/ AI)
Han *et al*, 2020 [38]	AlexNet	Cancer detection	Cancer presence annotated by 2 pathologists	286 WSIs from 68 patients	Leave one out cross val	None	Cancer classification at WSI level	AUC: 0.98
Ambrosini *et al*, 2020 [51]	CNN from scratch	Cribriform pattern detection	Patterns annotated by 2 pathologists	128 WSIs	8-fold cross val	None	Cribriform pattern detection for biopsies	$\overline{AUC}$: 0.8
Bukhari *et al*, 2021 [57]	ResNet 18, 34 and 50	Cancer or hyperplasia classification	Labeled by two pathologists	640 WSIs	162 WSIs	None	Cancer/hyperplasia detection at tile level (ResNet-50)	F1: 1<br>ACC: 0.995
							Cancer/hyperplasia detection at tile level (ResNet-34)	F1: 1<br>ACC: 0.98
							Cancer/hyperplasia detection at tile level (ResNet-18)	F1: 0.974<br>ACC: 0.971
Pinckaers *et al*, 2021 [33]	ResNet-34 (MIL)	Cancer detection	Train: clinical report<br>Test: 3 specialists	5209 biopsies	535 biopsies	205 biopsies	Cancer detection with MIL on biopsy	AUC: 0.99 (IV)<br>0.799 (EV)
	Streaming CNN						Cancer detection with Streaming on biopsy	AUC: 0.992 (IV)<br>0.902 (EV)
Perincheri *et al*, 2021 [49]	Paige Prostate	PaigeProstate evaluation	Clinical diagnosis of cancer suspicion	Pre-trained [31]	Pre-trained [31]	1876 biopsies from 116 patients	Judge Paige classification	110 patients were rightly classified out of 118<br>Discrepancy of 80 out of 1876 cores
Da Silva *et al*, 2021 [48]	PaigeProstate	PaigeProstate evaluation	Benign/cancerous/suspicious annotations by 2 pathologists	Pre-trained [31]	Pre-trained [31]	600 biopsies from 100 patients	Malignancy classification for biopsies	Sens: 0.99<br>Spec: 0.93
							Malignancy classification for patients	Sens: 1<br>Spec: 0.78

Raciti *et al*, 2022 [47]	PaigeProstate	Improvement of pathologists using AI	18 pathologists, with 2 genitourinary specialists	Pre-trained [31]	Pre-trained [31]	610 biopsies	Cancer detection for patients	Sens: 0.974 (mean increase of 8%)<br>Spec: 0.948<br>AUC: 0.99
Krajnansky *et al*, 2022 [58]	VGG16-model with explanation maps	Improve explainability of neural networks	NA	156 biopsies from 262 WSIs	10 biopsies from 87 WSIs	None	Malignancy detection for biopsy	FROC: 0.944
							Malignancy detection for patients	AUC: 1
Tsuneki *et al*, 2022 [53]	EfficientNetB1 pretrained on colon	Cancer detection on needle and TUR-P biopsies	Cancer presence by 3 pathologists	1182 needle biopsies	1244 TUR-P biopsies<br>500 needle biopsies	767 slides	Cancer detection in classic and TUR-P biopsies	AUC: 0.967 (IV) & 0.987 (EV)<br>AUC (TUR-P): 0.845
	EfficientNetB1 pretrained on ImageNet							AUC: 0.971 (IV) & 0.945 (EV)<br>AUC (TUR-P): 0.803
Tsuneki *et al*, 2022 [54]	EfficientNetB1 pretrained on ImageNet	Cancer detection on needle and TUR-P biopsies	Cancer presence by 3 pathologists	1060 TUR-P biopsies	500 needle biopsies<br>500 TUR-P	768 slides	Cancer detection in classic and TUR-P biopsies	AUC: 0.885 (IV) TUR-P<br>AUC: 0.779 (IV) & 0.639 (EV) biopsy
	EfficientNetB1 pretrained on colon							AUC: 0.947 (IV) TUR-P<br>AUC: 0.913 (IV) & 0.947 (EV) biopsy
Chen *et al*, 2022 [59]	DenseNet	Cancer tissue malignancy classification	Annotations of malignancy by 1 pathologist	29 WSIs	3WSIs	None	Classification of tissue malignancy	AUC: 0.98 (proposed method)<br>AUC: 0.90 (only DenseNet-121)

IV: Internal Validation. EV: External Validation. TMA: Tissue MicroArray. CNN: Convolutional Neural Network. MIL: Multiple Instance Learning. TURP: TransUrethral Resection of Prostate.
AUC: Area Under the Curve. FROC: Free Receiver Operating Characteristic. ACC: Accuracy. F1: F1-score. Sens: Sensitivity. Spec: Specificity.
$\overline{metric}$ implies the mean of this metric (e.g. $\overline{AUC}$)

Supplementary Table S6. Articles focusing on Gleason grading

First author, year Reference	DL architecture(s)	Methodology for/to	Ground-truth	Training cohort	Internal validation (IV)	External validation (EV)	Aim	Results
Källén et al, 2016 [63]	OverFeat	Classify patches on	TCGA annotation or pathologist classification	TCGA	10-fold cross val	213 WSIs	GP tile classification	ACC: 0.81
							Classify WSIs with majority GP	ACC: 0.89

		benign, grade 3/4/5						
Jimenez Del Toro *et al*, 2017 [74]	GoogleNet	High VS Low grade classification	Extracted from pathology reports	141 WSIs	47 WSIs	None	High VS Low grade classification	ACC:0.735
Arvaniti et al, 2018 [21]	MobileNet + classifier	Benign, low- or high-grade patient classification	Annotation of cancerous regions with GP	641 TMAs	245 TMAs	None	TMA grading	qKappa: 0.71/0.75 (0.71 pathologists)
							Tile grading	qKappa: 0.55/0.53 (0.67 pathologists)
Poojitha et al, 2019 [64]	CNNs	Correctly grade GP 2-3-4-5	GP defined on samples by expert	80 samples	20 samples	None	GP estimation at tile level (GP 2 to 5)	F1: 0.97
Nagpal et al, 2019 [73]	InceptionV3	Patient Gleason grading	GG on WSIs and GP annotations by 4 pathologists	1159 WSIs	331 WSIs	None	Gleason Grouping	ACC: 0.7
							High/low grade classification (GG 2, 3 or 4 as threshold)	µAUC: 0.95
							Survival analysis according to Gleason	c-index: 0.65 (0.69 for experts)<br>HR: 1.38
Silva-Rodriguez et al, 2020 [52]	Custom CNN	Detect cribriform patterns and characterize GG	GP annotations<br>WSIs GG and cribriform pattern presence	155 WSIs	5-fold cross-val	703 patches (EV)	Patches Gleason patterns	µF1: 0.713 (IV) & 0.57 (EV)<br>qKappa: 0.732 (IV) & 0.64 (EV)
							Gleason grouping WSIs	qKappa: 0.81 (0.77 with [20] method)
							Cribriform pattern detection on patches	AUC: 0.822
Otalora et al, 2021 [65]	MobileNet-based CNN	GG classification with strong or weak annotations	GP annotations for TMAs<br>WSIs GG	641 TMAs<br>255 WSI	245 TMAs<br>46 WSI	None	Gleason grouping	wKappa: 0,52
Hammouda et al, 2021 [78]	CNNs	GG (multiple resolution)	Ground truth defined in the Panda dataset	712 WSIs	96 WSIs	None	GP at tile level	$\overline{F1}$: 0.76 (same as ResNet50)
							Gleason grouping	$\overline{F1}$: 0.6
Marini et al, 2021 [66]	Custom CNN	GS of TMAs and WSIs	GP annotations for TMAs<br>GS on WSIs	641 TMAs<br>255 WSI	245 TMA<br>46 WSI	None	GP at tile level	qKappa: 0.66
							GS at TMA level	qKappa: 0.81
Marron-Esquivel *et al*, 2023	DenseNet121	Comparing inter-observer	Annotations by at least 2 pathologists	15020 patches	2612 patches	None	Patch level GP classification	qKappa: 0.826

[77]		variability (qKappa: 0.69) to DL models when grading		102324 patches (PANDA WSIs then fine-tuned)				qKappa: 0.746
Ryu et al, 2019 [72]	DeepDx Prostate	GG classification of patients	Annotation of benign, GP by 3 pathologists	1133 WSIs	700 WSIs	None	Gleason grouping	qKappa: 0.907
Karimi et al, 2019 [61]	Custom CNN	Patch classification of malignancy and GP	Annotations of benign and GP by 2 or 4 pathologists	247 TMAs	86 TMAs	None	Malignancy at tile level	Sens: 0.86<br>Spec: 0.85
							Patch GP 3 vs. 4/5 at tile level	Sens: 0.82<br>Spec: 0.82
Nagpal et al, 2020 [35]	Xception	Patient classification of malignancy and GG	Annotation of GP<br>Gland annotation	524 WSIs	430 WSIs	322 WSIs	Malignancy detection at WSI level	AUC: 0.981<br>Agreement: 0.943
							GG1-2 vs. GG3-5	AUC: 0.972<br>Agreement: 0.928
Pantanowitz et al, 2020 [36]	IBEX	Cancer, GS, GP 5 and PNI classification	GP annotations by 3 pathologists	549 WSIs	2501 WSIs	1627 WSIs	Cancer detection on WSIs	AUC: 0.997 (IV) & 0.991 (EV)
							Low vs. high grade (GS 6 vs GS 7-10)	AUC: 0.941 (EV)
							GP3/4 vs. GP5	AUC: 0.971 (EV)
							Perineural invasion detection	AUC: 0.957 (EV)
Ström et al, 2020 [37]	InceptionV3 model	Detect cancer in slides	Malignancy and GG on WSIs by 1 pathologist	6935 slides	1631 biopsies	330 biopsies	Malignancy of slides	AUC : 0.997 (IV)<br>0.986 (EV)
							Gleason grouping	Kappa : 0.62
Li et al, 2021 [39]	Weakly supervised VGG11bn	Low VS high grade classification	Pathology report	661 patients (13115 WSIs)	169 patients (7114 WSIs)	79 WSIs (19 benign, 60 malignant)	Malignancy of WSIs	AUC : 0.982 (IV) & 0.994 (EV)
							Low vs. high grade at WSI level	Kappa : 0.818<br>Acc : 0.927
Kott et al, 2021 [62]	ResNet	Benign and GP identification	GP annotations by 1 pathologist	85 WSIs	5-fold cross-validation	None	Malignancy detection at tile level	AUC : 0.83<br>ACC : 0.85 for fine-tuned detection
							GP classification at tile level	ACC : 0.85<br>Sens : 0.83<br>Spec : 0.94
Marginean et al, 2021	CNN	Cancer detection and	Annotation of GP by 2 pathologists		21 patients (37 biopsies)	None	Cancer area detection	Sens : 1<br>Spec : 0.68

[79]		Gleason grouping		174 patients (698 biopsies)			Grade group classification	$\overline{Kappa}$: 0.6
Jung et al, 2022 [75]	DeepDx Prostate	Cancer detection and Gleason grading by a commercially developed software	GS and GG determined by 3 pathologists	Pre-trained	Pre-trained	593 WSIs	Correlation with reference pathologist (pathology report comparison)	Kappa : 0.654 (0.576)<br>qKappa : 0.904 (0.858)
Silva-Rodriguez et al, 2022 [40]	VGG16	Gleason grading on a small dataset with weak supervision	GS attributed to each slide during diagnosis	252 WSIs	98 WSIs	None	Cancer detection at tile level	AUC: 0.979
							GS at tile level	AUC: 0.899
							GP at tile level	$\overline{F1}$: 0.65 (0.75 prev. Paper)<br>qKappa: 0.655
Bulten et al, 2022 [76]	Evaluation of multiple algorithms (PANDA challenge)	GG classification of patients with weak labels	Train: pathological reports<br>Validation: 4 pathologists reviewed GG	10 616 WSIs	545 WSIs	741 patients (EV1)<br>330 patients (EV2)	Gleason Grouping	qKappa: 0.868 (EV2)<br>qKappa: 0.862 (EV1)
Li et al, 2018 [68]	Multi-scale U-Net	Segment WSI patches	Annotations by 3 pathologists	187 tiles	37 tiles	None	Segment stroma, glands benign or not	IoU: 0.755<br>(0.750 classic U-Net)
							Segment stroma, benign and GP 3/4	IoU: 0.658<br>(0.644 classic U-Net)
Li et al, 2019* [29]	R-CNN	Segment stroma, benign, low- and high-grade tissue	Annotations by 2 pathologists	513 WSIs	5 fold cross val	None	Segment stroma, benign, low- and high-grade tissue	IoU: 0.79 (mean amongst classes)
Bulten et al, 2019* [28]	U-Net	Segment GP on slides	GG of patients on slides	62 WSIs	40 WSIs	20 WSIs	Segment benign and GP	IoU: 0.811 (IV) & 0.735 (EV)<br>F1: 0.893 (IV) & 0.835 (EV)
Lokhande et al, 2020 [69]	FCN8 based on a ResNet50	Segment areas of different GP	GP annotations by 3-6 pathologists	172 TMAs	72 TMAs	None	Segmentation between benign/grade 3/4/5	Dice: 0.74 (average amongst all classes)

Li et al, 2018 [71]	Multi-Scale U-Net-based CNN	Stroma, benign, GP segmentation	Tissue & GP annotations made by 4 pathologists	50 patients	20 patients	None	See if EM improves performances of multi-scale U-Net	$\overline{IoU}$: 0.35 (U-Net)<br>$\overline{IoU}$: 0.49 (EM-adaptative 30%)
Bulten et al, 2020 [41]	Unet extended	Cancer detection and GG classification	Train: pathology reports<br>Validation: 3 pathologist consensus	5209 biopsies from 1033 patients	550 biopsies from 210 patients	886 cores	Malignancy detection at WSI level	AUC: 0.99 (IV) & 0.98 (EV)
							GG >2 detection	AUC: 0.978 (IV) & 0.871 (EV)
					100 biopsies	None	Gleason grouping	qKappa: 0.819 (general pathologists)<br>0.854 (DL) & 0.71 (EV)
Hassan et al, 2022 [70]	ResNet50	Segment background, stroma, benign and GP-tissue	Area annotation made by pathologists	18264 WSIs	3251 WSIs	None	Tissue segmentation for GG presence	$\overline{IoU}$: 0.48<br>$\overline{F1}$: 0.375
Lucas et al, 2019 [67]	Inception V3	Differentiate GP3 & 4 and non-atypical tissue	Pixel annotations by 3 pathologists	72 WSIs	24 WSIs	None	Malignancy detection at pixel level	Sens: 0.90<br>Spec: 0.93
							GP3 & GP4 segmentation at pixel level	Sens: 0.77<br>Spec: 0.94

*Articles already in Supplementary Table 3 for segmentation tissue performances. Double line separates classification (above) from segmentation algorithms (below).
IV: Internal Validation. EV: External Validation. TMA: Tissue MicroArray. CNN: Convolutional Neural Network. GG: Gleason ISUP Group, GS: Gleason Score, GP: Gleason Pattern.
AUC: Area Under the Curve. ACC: Accuracy. F1: F1-score, combination of precision and recall. IoU: Intersection over Union. q/wKappa: quadratic/weighted Cohen Kappa. Sens: Sensitivity. Spec: Specificity. $\overline{metric}$ implies the mean of this metric (e.g. $\overline{AUC}$)

Supplementary Table S7. Articles focusing on clinical outcome prediction

First author, year Reference	DL architecture(s)	Methodology for/to	Ground-truth	Training cohort	Internal validation (IV)	External validation (EV)	Aim	Results
Kumar et al, 2017 [81]	CNNs	Predicting recurrence	Annotations of nuclear boundaries	160 TMAs	60 TMAs	None	Nucleus detection for tile selection	ACC: 0.89

			Recurrence status (yes or no)				Recurrence prediction	AUC: 0.81(DL) & 0.59 (clinical data)
Ren et al, 2018 [83]	AlexNet + LSTM	Predict recurrence with also genomic data	RFS (Recurrence Free Survival)	271 patients	68 patients	None	Predict RFS	HR: 5.73 (5.06 w\o genomics)
Ren et al, 2019 [88]	CNN + LSTM	Predict recurrence	Biological RFS	268 WSIs	67 WSIs	None	Develop a survival model	HR: 7.10 when using image features
Leo et al, 2021 [87]	Segmentation-based CNNs	Prediction of prognostic based on cribriform pattern area (CAI)	BCR (BioChemical Recurrence) Annotations of cribriform patterns	70 patients	NA	679 patients	Annotate cribriform patterns	Pixel TPV: 0.94<br>Pixel NPV: 0.79
							Annotate lumen regions	Pixel TPV: 0.94<br>Pixel TNV: 0.97
							Prognosis classification using CAI	Univariable HR: 1.31<br>Multivariable HR: 1.66
Wessels et al, 2021 [84]	xse_ResNext34	Prediction of lymph node metastasis (LNM)	LNM positive or negative	118 patients	110 patients	None	Predict LNM based on initial RP slides	AUC: 0.69
Esteva et al, 2022 [85]	ResNet	Prediction of metastasis, survival. Addition of clinical variables	Clinical data for every outcome (binary) depending on time frames	4524 patients	1130 patients	None	Distant metastasis at 5 years (5Y) and 10 years (10Y)	AUC: 0.837 (5Y) (0.779 only images)<br>AUC: 0.781 (10Y) (0.728 only images)
							Prostate cancer specific survival	AUC: 0.765 (0.766 only images)
							Overall survival 10 years	AUC: 0.652 (0.587 only images)
Pinckaers et al, 2022 [82]	ResNet50	Prediction of patient prognosis	Biochemical recurrence or events indicating it (metastasis, death…)	503 patients	182 patients	204 patients	Univariate analysis to see if biomarker computed with DL is predictive	OR: 3.32 (IV)<br>HR: 4.79 (EV)
Liu et al, 2022 [86]	10-CNN ensemble model	Predict if a benign biopsy is from a man that has PCa or not	Diagnostic made by pathologist on all biopsies	9 192 benign biopsies from 1211 patients	2851 WSIs	None	Predict if slide belongs to cancer patient or no	AUC: 0.727
							Predict if patient has cancer or no, depending on a benign slide	AUC: 0.739
Huang et al, 2022 [82]	NA	Predicting recurrence of	BCR (3 year follow up)	243 patients	None	173 patients	Predict 3 Year recurrence	AUC: 0.78

		prostate cancer after RP						
Sandeman et al, 2022 [42]	Handcrafted CNN (AIForIA)	Cancer detection and GP to develop a survival model	Annotation of benign and GP tissue by 2 pathologists	331 patients	391 patients	126 patients (146 biopsies)	Detection of malignant vs benign	AUC: 0.997
							Grade grouping	ACC: 0.67 Cohen kappa: 0.77
			RALP outcome data				Prediction outcome from DL calssification	HR: 5.91

IV: Internal Validation. EV: External Validation. TMA: Tissue MicroArray. CNN: Convolutional Neural Network. RP: Radical Prostatectomies. LSTM : Long-Short Term Memory network. TMA: Tissue MicroArray.
AUC: Area Under the Curve. ACC: Accuracy. wKappa: weighted Cohen Kappa. HR: Hazard Ratio. OR: Odds Ratio. TPV: True Positive Value. TNV: True Negative Value. $\overline{metric}$ implies the mean of this metric (e.g. $\overline{AUC}$)

Supplementary Table S8. Articles focusing on genomic signatures prediction

First author, year Reference	DL architecture(s)	Methodology for/to	Ground-truth	Training cohort	Internal validation (IV)	External validation (EV)	Aim	Results
Schaumberg et al, 2018 [92]	ResNet50	Predicting SPOP mutations	Presence or not of SPOP mutations	177 patients	None	152 patients	SPOP mutation prediction	AUC: 0.74 (IV) AUC: 0.86 (EV)
Schmauch et al, 2020 [89]	HE2RNA	Predict RNA seq expression	RNA-seq signatures	8725 patients (Pan cancer)	5-fold cross val	None	Prediction of gene signatures specific to prostate cancer	PC: 0.18 (TP63) 0.12 (KRT8 & KRT18)
Chelebian et al, 2021 [91]	CNN from [34] fine-tuned	Inference of spatial transcriptomics	Two pathologists reviewed Visium ST data	Pre-trained ([33])	7 WSIs	None	Correlation between clusters identified with AI and spatial transcriptomics	No global metric is given
Dadhania et al, 2022 [93]	MobileNetV2	Predicting ERG gene fusion	ERG-rearranged or no status	261 patients	131 patients	None	ERG gene rearrangement status prediction	AUC: 0.82 to 0.85 (depending on resolution)
Weitz et al, 2022 [90]	NA			278 patients	92 patients	None	CCP gene expression prediction	PC: 0.527
							BRICD5 expression prediction	PC: 0.749

Predict RNA seq expression	RNA-seq signatures (50 clusters of co-expressed genes)		SPOPL expression prediction	PC: 0.526

IV: Internal Validation. EV: External Validation. CNN: Convolutional Neural Network. AI: Artificial Intelligence. CCP: Cell Cycle Progression. AUC: Area Under the Curve. PC: Pearson Correlation
